# Reasoning with Linear Orders: Differential Parietal Cortex Activation in Sub-Clinical Depression. An fMRI Investigation in Sub-Clinical Depression and Controls

**DOI:** 10.3389/fnhum.2014.01061

**Published:** 2015-01-19

**Authors:** Elanor C. Hinton, Richard G. Wise, Krish D. Singh, Ulrich von Hecker

**Affiliations:** ^1^Clinical Research and Imaging Centre, University of Bristol, Bristol, UK; ^2^Cardiff University Brain Research Imaging Centre (CUBRIC), School of Psychology, Cardiff University, Cardiff, UK; ^3^School of Psychology, Cardiff University, Cardiff, UK

**Keywords:** fMRI, sub-clinical depression, reasoning

## Abstract

The capacity to learn new information and manipulate it for efficient retrieval has long been studied through reasoning paradigms, which also has applicability to the study of social behavior. Humans can learn about the linear order within groups using reasoning, and the success of such reasoning may vary according to affective state, such as depression. We investigated the neural basis of these latter findings using functional neuroimaging. Using BDI-II criteria, 14 non-depressed (ND) and 12 mildly depressed volunteers took part in a linear-order reasoning task during functional magnetic resonance imaging. The hippocampus, parietal, and prefrontal cortices were activated during the task, in accordance with previous studies. In the learning phase and in the test phase, greater activation of the parietal cortex was found in the depressed group, which may be a compensatory mechanism in order to reach the same behavioral performance as the ND group, or evidence for a different reasoning strategy in the depressed group.

## Introduction

A fundamental ability in both humans and animals is the capacity to flexibly learn new information and to recall and manipulate that information for future use (Simons and Spiers, 2003; Manns and Eichenbaum, 2006). Indeed, both humans and animals can flexibly make novel inferences from the information provided (Dickins, 2005; Vasconcelos, 2008). This process is often studied through linear-order reasoning paradigms (Potts, 1972; Sternberg, 1980), in which participants learn A > B and B > C; evidence of reasoning occurs when they can rearrange the incoming information into a coherent representation, or mental model, in order to infer that A > C. This type of reasoning is not purely an abstract cognitive process, however, but one which has applications in the environment; for example, animals use this type of processing to learn their place in the social order of their groups (Hogue et al., 1996; Paz-Y-Miño et al., 2004). Humans can learn about rank orders within groups of people using linear-order reasoning, and the success of such reasoning may depend on affective state, particularly, sub-clinical depression (Sedek and Von Hecker, 2004). Previous research has found dysfunctions in the frontoparietal network in depressed participants [for an overview see Brzezicka (2013)]. In particular, Thomas and Elliott (2009), as well as Hugdahl et al. (2004) found in their depressed participants that reduced parietal activity was associated with impaired performance in mental arithmetic tasks, as well as hyperactivity was associated with intact performance, leading these authors to conclude that normal performance in depression is associated with enhanced cortical, in particular parietal, function during reasoning. In this study, we use functional MRI to investigate how brain activation during execution of a different reasoning task, that is, linear-order construction, might be altered in the brain, especially in parietal cortical areas, when individuals are in a state of sub-clinical depression.

There is an increasing literature on the neural basis of linear order, or transitive, reasoning [e.g., Christoff et al. (2001), Goel and Dolan (2001, 2003, 2004), Acuna et al. (2002), Knauff et al. (2002), Fangmeier et al. (2006), Greene et al. (2006), Monti et al. (2007), Van Opstal et al. (2008), Wendelken et al. (2008)]. Studies to date have largely taken an abstract form in the tasks employed to reveal the underlying brain activation of making inferences. A review of the above literature demonstrates that a “network” of brain regions subserve reasoning, including the hippocampus, parietal, and prefrontal cortices. Knauff et al. (2002) found that an occipital–parietal–frontal network was activated during relational reasoning, which includes areas in the visuospatial system. In line with this research, we suggest that spatial processing of relations is paramount to processing orders or hierarchies in order to solve reasoning problems (Leth-Steensen and Marley, 2000). Specifically, the present study will look into the areas of intra-parietal sulcus, inferior parietal lobe (BA 40), and posterior parietal lobe (BA 7) as earlier work has suggested that these regions might be involved in tasks involving spatial and numerical operations, as well as working memory [e.g., D’Esposito et al. (1998), Sakai et al. (1998), Pinel et al. (2001)], and, more specifically, in the spatial operations during transitive inference (Goel and Dolan, 2001; Acuna et al., 2002; Knauff et al., 2002). Furthermore, the role of the prefrontal cortex (PFC) in reasoning has been highlighted in studies of relational complexity and integration (Christoff et al., 2001; Acuna et al., 2002; Kroger et al., 2002; Wendelken et al., 2008).

In the present study, rather than employing abstract symbols in the task, we focus on more naturalistic linear-orders regarding relationships within small sets of people. During functional magnetic resonance imaging (fMRI), participants learned a series of pairwise information, such as “Andrew is taller than Brian,” “Brian is taller than Colin,” and “Colin is taller than David.” Evidence suggests that people spontaneously rearrange the three presented pairs of information and integrate them into a coherent mental model (≥“taller”): A > B > C > D, most likely involving spatial representations (Huttenlocher, 1968; Waltz et al., 1999). After the learning phase, test queries were asked about all possible pairs of names, such as the three presented ones, i.e., A/B, B/C, C/D, and also queries about those relations that were not presented during learning, such as A/C, B/D (an inference spanning two distance steps along the assumed mental model), and A/D (involving two inferences, and corresponding to three distance steps along the model).

There is some evidence to suggest that transitive reasoning is affected by sub-clinical depression (Sedek and Von Hecker, 2004). Such reasoning deficits may lie at the heart of some cognitive problems found in those with depression, such as loss of creativity and inferior ability to solve problems in the social domain (Gotlib and Hammen, 1992; Marx et al., 1992; von Hecker and Sedek, 1999). Depressed participants showed inferior performance as compared to non-depressed (ND) controls in the linear-order task as described above, especially concerning the inferred pairs (Sedek and Von Hecker, 2004, Exp. 1, 3, and 4). The authors suggested that while ND individuals might create the comprehensive model A > B > C > D spontaneously during learning, depressed individuals might not do so (or not be successful in doing so), but engage in reasoning more upon particular queries during the test phase, this resulting in a less efficient processing overall. The present hypothesis, therefore, is that compared to those without depression, individuals in depressed states may show higher indices of brain activation in the spatial areas supporting transitive reasoning as described above, when tested on queries of any pair distance across the linear-order A > B > C > D.

## Materials and Methods

### Participants

Female participants were recruited into this study on the basis of their score on the Beck depression inventory-II (Beck et al., 1996). Only females were recruited for this study, as there is a greater prevalence of depression in females (Nolen-Hoeksema, 2002). Participants attended one or two sessions. In the first session, participants were given the BDI and CED depression scales, and the operation span (OSPAN) and digital symbol substitution test (DSST) tasks (see below for details). Participants who fitted the BDI criteria for the ND or D groups in the first session were asked to attend a second session 1 week later. In the second session, participants were given both depression scales again. If their scores allowed them to remain in their original group classification, they immediately took part in the imaging phase. If not, the reasons for them not continuing onto the imaging session were given, and they were thanked and debriefed. For the ND group, those with a score of 5 or below, on two occasions 1 week apart, were chosen (*n* = 17). Those with a score of 13 or above, on two occasions 1 week apart, were included in the mildly depressed (D) group (*n* = 15). Participants were given a second depression scale (Center for Epidemilogic Studies Depression scale, CES-D, Radloff, 1977) in the second session, on which participants had to get a score of 16 or above to remain in the D group.

Data from three participants from the D group and three from the ND group had to be excluded from the analysis either due to excessive movement in the scanner or misunderstanding the task instructions. Twenty-six participants remained in the analysis: 14 in the ND group and 12 in the D group. Table 1 summarizes the group demographics. All participants indicated that they were right-handed, none had any history of psychiatric or neurological disorders, and none were currently taking psychotrophic medications. All participants gave informed consent. This study was approved by the Cardiff School of Psychology Ethics Committee.

**Table 1:** **Participant information**.

	Non-depressed group (ND)	Depressed group (D)
*N*	14	12
Age (years)	22.6 (3.9)	22.7 (5.7)
BDI-II at time 1	2.3 (1.6)	16.6 (2.9)*
BDI-II at time 2 (imaging)	0.9 (1.1)	20.6 (7.0)*
CES-D at time 2 (imaging)	1.7 (2.2)	23.9 (6.9)*
DSST score	48.4 (6.8)	48.7 (10.1)
OSPAN words score (WM)	11.9 (6.3)	12.0 (7.8)
OSPAN maths score	37.2 (5.2)	37.4 (2.7)

(SD given in brackets) *indicates a significant difference at the level of *p* < 0.05 using an independent groups *t* test.

### Behavioral tasks and design

**Figure 1:**
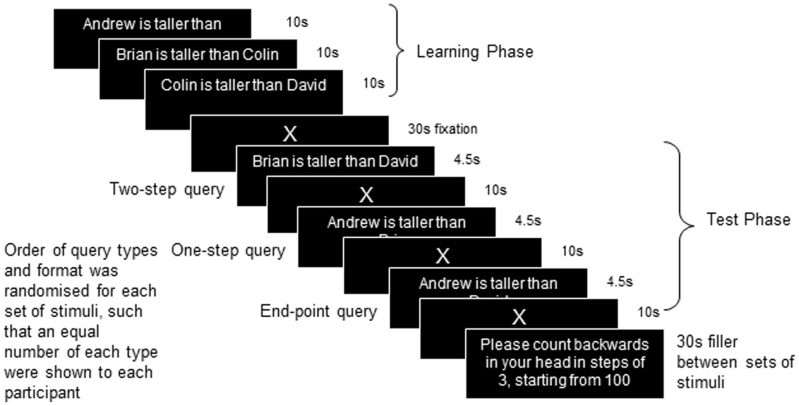
**Design of the study**.

During the fMRI, a mixed block/sparse event-related design was used to present the linear-order reasoning task (Figure 1). As described above, participants were shown information regarding the relationships between four people (A > B > C > D), upon which they were then tested. In the initial learning phase, presented as a block, participants were sequentially shown three sentences for 10 s each, followed by 30 s of fixation to a cross (X). Participants were asked to remember the names and the relationships between them, e.g., of one set (1) Andrew is taller than Brian (A > B) (2) Brian is taller than Colin (B > C) (3) Colin is taller than David (C > D). Other relational terms included “older,” “richer,” “smarter,” “braver,” and “faster” (18 in total). Relational pairs were presented in equal numbers of one of two order types: (i) where the pairs are presented in the order in which they appear in the putative model (e.g., A > B, B > C, C > D), or (ii) where the relations appear in a different order to the model (e.g., B > C, A > B, C > D), in order to assess whether the latter required differential brain activity to support the greater cognitive demands to support the integration of pairs. A test phase followed in which a query sentence was presented for 4.5 s followed by 10 s of fixation to allow the BOLD response to return to baseline between events. Three query sentences were presented in each test phase: One sentence was randomly chosen from those presented in the learning phase (“one step” queries, A > B, B > C, or C > D, equivalent to one step (A to B) on the hypothetical mental model), one sentence was randomly chosen from “two-step” queries (A > C, B > D), and the end-point query was presented (A > D). The queries were either presented in correct or incorrect format (e.g., Andrew is taller than Brian, or Brian is taller than Andrew). Participants had to respond whether or not the query content was correct on the basis of the information learned about the group of people in the learning phase. Twelve sets of stimuli were presented in three imaging runs of four sets. The format of the test phase queries were pseudo-randomized such that over the course of the three runs there were an equal number (12) of each type of query (one step, two step, and end point), and an equal number of correctly (6), and incorrectly presented trials (6). Total scan time was approximately 30 min, followed by an anatomical brain scan for a further 10 min.

Data from the reasoning task were analyzed using ANOVA. The dependent measures were the percentage of correct responses and response time (within the 4.5 s window) to each query type (one step, two step, and end point). Following the imaging session, participants were given a post-imaging questionnaire, designed to ascertain how participants reported doing the task. Participants were also given an OSPAN task (Turner and Engle, 1989) as a measure of working memory capacity, and the DSST, a subset of the WAIS-R (Wechsler, 1981), as a measure of processing speed. There were no significant differences between the groups on these two control measures (see Table 1 for means; OSPAN *t* = −0.031, *p* > 0.05; DSST *t* = −0.071, *p* > 0.05), so any differences found on the reasoning task cannot be attributed to differences in processing speed or working memory capacity.

### Image acquisition

Anatomical and functional images were acquired at the Cardiff University Brain Research Imaging Center (CUBRIC), using a General Electric Excite-HDx 3 T MRI scanner. Functional images were collected using a gradient-echo echo-planar pulse sequence (TE = 35 ms; TR = 2500 ms; flip angle = 90°; acquisition matrix = 64 × 64; field of view (FOV) 64 × 64; in plane resolution 3.75 mm). The volumes covered the whole brain in 37 slices (thickness 3.8 mm) and were acquired in line with the anterior commissure/posterior commissure line. A total of 684 volumes were acquired for each participant in 3 sessions of 228 volumes each. In each run of 228 volumes, 3 sets of stimuli were presented. For each set (as described above, see Figure 1 for presentation timing of one set of stimuli), a learning phase of 3 premise pair sentences (e.g., A > B, B > C, C > D), each presented for 10 s, was followed by a fixation cross for 30 s. The test phase then immediately followed with 3 test queries (4.5 s each), each followed by 10 s fixation. A filler task (counting backwards for 30 s) was given to participants between each set in order to reduce possible interference between sets of relations. This results in 12 block scans for analysis of the learning phase, and 12 one-step, 12 two-step, and 12 end-point test queries for analysis of the test phase. The timing of the program in presentation was designed such that the test queries were not presented until a pulse had been received by the scanner. This ensured that the task was always in synchrony with the scanner. Finally, a high-resolution T1-weighted FSPGR anatomical image was acquired (TR = 7.9 s; TE = 3 ms; inversion time = 450 ms; flip angle = 20°; acquisition matrix 256 × 256 × 176; FOV 256 × 256 × 176, resulting in 1 mm isotropic voxels).

### Image analysis

Data was analyzed using the FSL package from FMRIB, University of Oxford (http://www.fmrib.ox.ac.uk/fsl/). For each participant, data were acquired in three runs. At the first level, each run was pre-processed and analyzed separately, using the following stages: motion correction using MCFLIRT (Jenkinson et al., 2002), non-brain removal using BET (Smith, 2002), spatial smoothing using a Gaussian kernel of FWHM 5 mm, mean-based intensity normalization of all volumes, and high-pass temporal filtering. Time-series statistical analysis was carried out using FILM with local autocorrelation correction (Woolrich et al., 2001). The first level modeled nine explanatory variables (EVs) for learning phase order 1 and 2, the filler task between sets, one-step, two-step, and end-point test queries presented in the correct or incorrect format. Contrasts compared: (1) learning phase to baseline, (2) the two different order of premises in the learning phase, (3) each test query type to baseline, (4) one-step to two-step queries, and (5) presented (one step) vs. inferred queries (two step and end point). At the second level, the separate runs were combined into a fixed analysis for each person, and then finally data from all participants was combined in a third level analysis for each contrast. Higher-level group analysis was carried out using a mixed effects group analysis – FLAME (stage 1 only) (Beckmann et al., 2003; Woolrich et al., 2004). *Z* statistic images were thresholded using Gaussian random field (GRF)-theory based maximum cluster thresholding with a corrected significance threshold of *p* = 0.05 (Worsley et al., 1992). Registration to high resolution and standard images was carried out using FLIRT (Jenkinson and Smith, 2001; Jenkinson et al., 2002).

The study was designed to examine differences between groups (ND and D) and between test relation types (one step, two step, and end point). For the learning phase data, contrasts examined (i) activation during the learning phase compared to baseline (fixation cross) between the two groups, and (ii) activation during the learning phase for each order of presented relations, using a whole-brain corrected cluster-based threshold (*z* > 2.3, *p* < 0.05). A subsequent analysis repeated (i), but for both groups together. When reporting data for both groups together a stricter threshold (*z* > 5) was chosen due to the large extent of activation found when simply comparing task to fixation baseline.

For the test phase data, only correctly answered trials were included in the analysis. This resulted in 5.3% of the total number of trials being excluded from the analysis. Contrasts examined (iii) each test query type compared to baseline across groups, (iv) previously presented queries compared to queries requiring inference, and most importantly (v) between group differences for each test query type (one step, two step, and end point). The MNI coordinate system is used in the results section when reporting the activation peaks.

## Results

### Behavioral data

#### Reasoning accuracy

**Figure 2:**
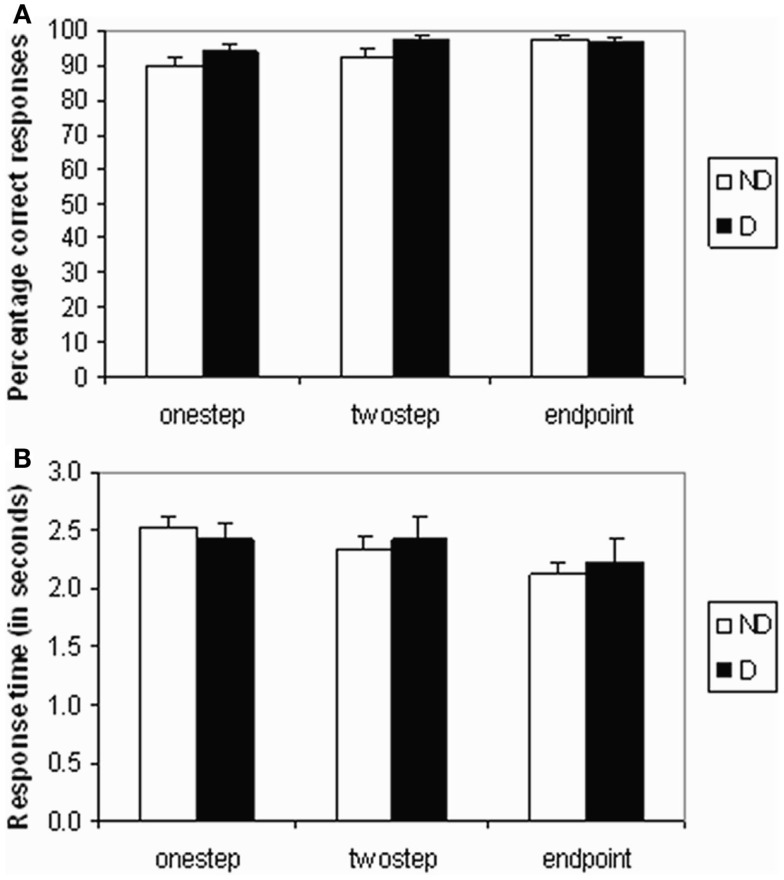
**Behavioral data**. **(A)** Mean accuracy scores (percentage of correct responses) and **(B)** mean response time in seconds for each test query type (one step, two step, or end point) and for each group (non-depressed or depressed).

The percentage of correct responses to the test queries is shown in Figure 2A. The main effect of pair distance (step) was significant (*F*_2,48_ = 5.061, *p* = 0.01), with accuracy increasing from one step queries to end-point queries. However, there were no significant differences in task accuracy between mood groups, between neighboring distances (one step/two step or two step/end point), or any interaction between group and pair distance.

#### Response times data

A significant stepwise decrease in reaction time was found across query types of increasing pair distance (*F*_2,48_ = 11.30, *p* < 0.001) – see Figure 2B. Pairwise comparisons showed that end-point queries needed significantly less time than two-step queries (*p* = 0.005), while the difference between one-step and two-step queries was not significant. There was also no significant difference between mood groups or any interaction between group and pair distance.

#### Questionnaire responses

All 26 participants reported, without prompting, that they had ordered the people in each set according to the relation specified between them, during the learning phase. Twenty-four out of 26 participants reported verbally rehearsing the correct order of the people in each set during the fixation between the learning and test phases; of the remaining participants, 1 reported using a purely visual strategy, and the other reported simply fixating on the cross.

### Neuroimaging data

#### Learning phase

No significant differences were found in the whole-brain analyses brain activation between D and ND groups, while the participants were learning the relations between the people in each group. Moreover, no significant differences were found according to the order of presenting the relational pairs. As such, the following results are reported including all 26 participants and both order types using a whole-brain corrected cluster-based threshold (*z* > 5, *p* < 0.05). A distributed network of areas was activated in association with the learning phase of the task relative to fixation (see Table S1 in Supplementary Material), including prefrontal and parietal cortex, hippocampus, as well as occipital cortex and cerebellum. (NB. *Post hoc* ROI analyses of the learning phase are presented below).

#### Test phase

First, to investigate the basic pattern of activation associated with the test phase queries, an average map of the activation found in association with each type of test query relative to fixation (one step, two step, end point) for both D and ND groups together is reported, which revealed a similar pattern across the query types. For summary purposes, Table S2 in Supplementary Material contains the results from all 26 participants together, using a whole-brain corrected cluster-based threshold (*z* > 5, *p* < 0.05).

**Figure 3:**
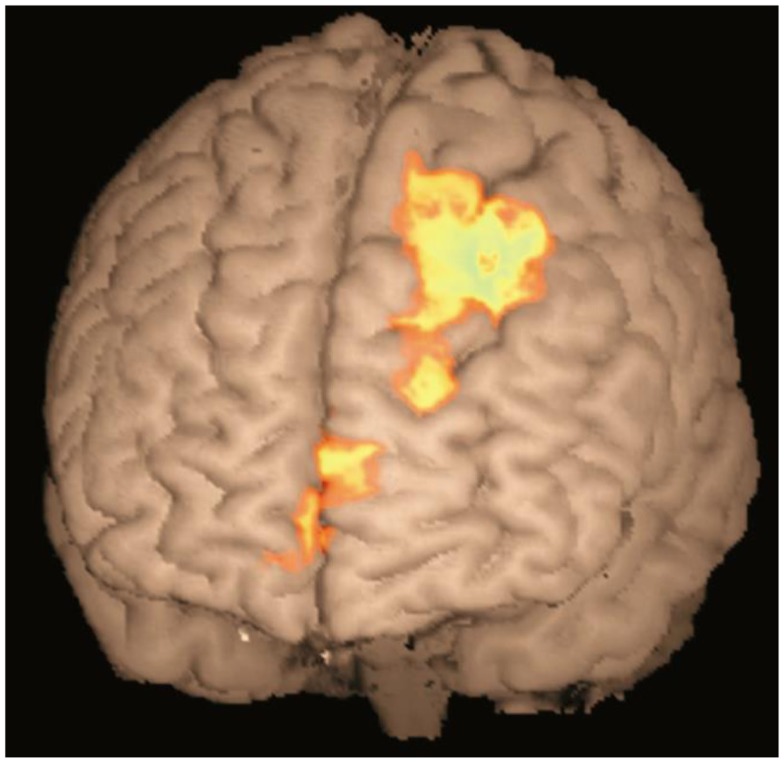
**Activation map for inferred queries compared to previously presented queries in ND group**. Activation shown in medial frontopolar cortex (BA 10, peak −4, 64, 14, *z* = 3.26) and superior frontal cortex (BA8, peak −26, 32, 50, *z* = 3.63) in the contrast between inferred queries (two step and end point) compared to previously presented queries (one step), in the non-depressed group only. Cluster-based threshold: *z* > 2.3,*p* < 0.05.

In order to examine, which areas were involved in making inferences, a further comparison was made between the response to test relations involving making an inference (two-step and end-point relations) and those involving one-step relations that would require recalling the previously presented information from the learning phase. A significant difference in brain activation between inferred and presented queries was found in the ND group only, using a whole-brain corrected cluster-based threshold (*z* > 2.3, *p* < 0.05). As shown in Figure 3, greater activation was found in the superior and medial frontal cortex in association with inferred queries (e.g., A > C, A > D) compared to the previously presented queries (e.g., A > B). The same regions were not significantly differentially activated in the depressed group for the same contrast. However, the direct comparison between groups did not reach significance [ND(inferred-presented) − D(inferred-presented)]. It is possible that the frontal cortex was activated more to inferred queries than presented queries in the depressed group as well, but that this difference in activation did not reach significance^1^.

**Figure 4:**
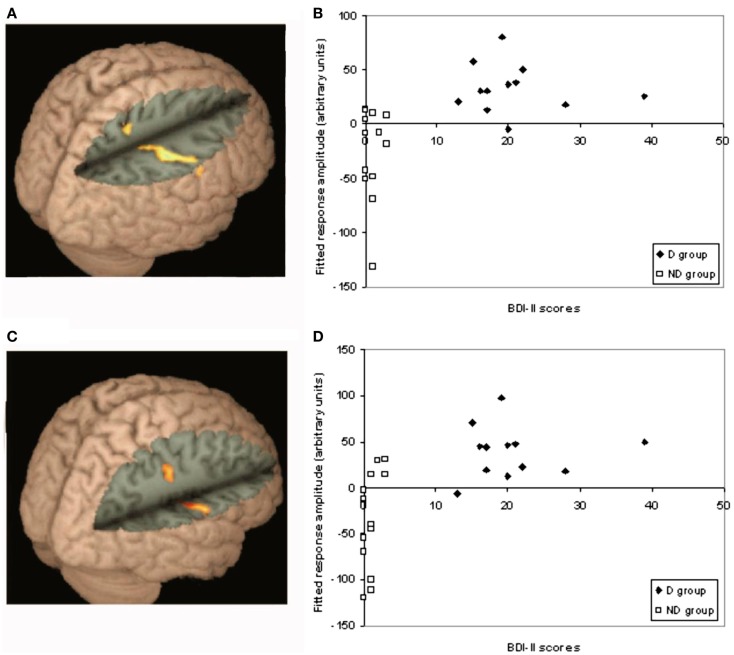
**Differences between D–ND groups for end-point and one-step queries**. This figure shows the significantly different pattern of activation found in the parietal lobe/post-central gyrus for end-point and one-step queries between groups (D–ND): **(A)** for end-point queries, foci were found in superior parietal cortex (26, −46, 60, *z* = 3.72), supramarginal gyrus/post-central gyrus (44, −26, 40, *z* = 3.76); **(B)** shows how activation in each group during end-point queries varied according to BDI-II score; **(C)** for one-step queries, foci were found in the parietal lobe (post-central gyrus 60, −12, 20, *z* = 3.95; 58, −1, 46, *z* = 3.87); **(D)** shows how activation in each group during one-step queries varied according to BDI-II score.

One of the key contrasts of interest in this study was to investigate differences in activation in response to the different test relations, relative to fixation, between the D and ND groups [D(test-fixation) − ND(test-fixation)]. Activation associated with each test query was analyzed between groups, using a whole-brain corrected cluster-based threshold (*z* > 2.3, *p* < 0.05). A significantly different pattern of activation was found in the parietal lobe/post-central gyrus for end-point and one-step queries between groups (D–ND), as shown in Figure 4A (end point), Figure 4C (one step). For end-point queries, foci were found in superior parietal cortex (26, −46, 60, *z* = 3.72), supramarginal gyrus/post-central gyrus (*x* = 44, *y* = −26, *z* = 40, *Z* = 3.76). For one-step queries, foci were found in the parietal lobe (post-central gyrus *x* = 60, *y* = −12, *z* = 20, *Z* = 3.95; 58, −1, 46, *z* = 3.87). Figure 4B (end point) and Figure 4D (one step) show how activity in these regions varies as a function of BDI-II score. These scatter-plots show that on average the ND group shows relative deactivation in these regions, whereas the D group show activation. A similar pattern of activation was found in the two-step contrast as for the other test query types (as shown in Table S2 in Supplementary Material above), and a D–ND difference was found for two-step queries in the same areas as for end-point and one-step queries after lowering the threshold slightly, suggesting that any difference between the groups did not quite survive the cluster threshold for two-step queries.

**Figure 5:**
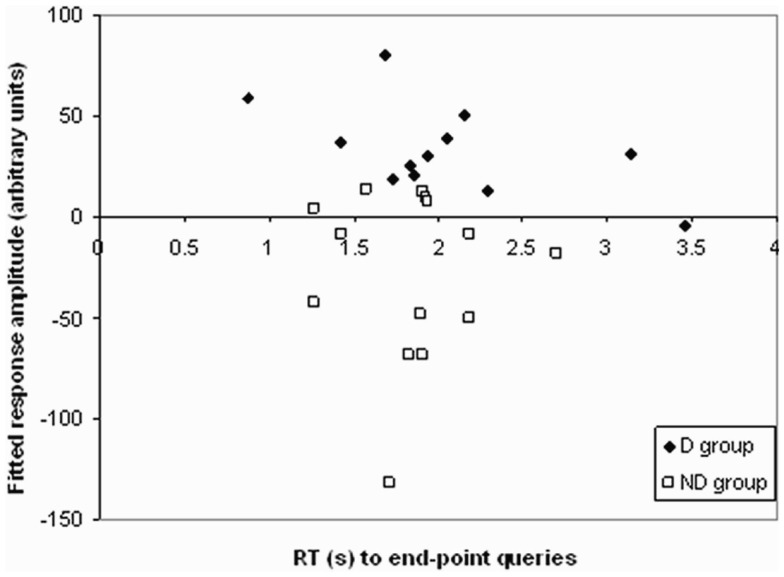
**Correlation in D group only between RT to end-point queries and activation in regions in the D–ND contrast**. A scattergram plotting the reaction time during end-point queries against activation in regions in the D–ND contrast in both groups. A significant negative correlation between response time, after checking for outliers (none were found) using the Tukey criterion (Clark-Carter, 2004, Chapter 9), and activation during end-point trials was found in the D group (*r* = −0.579, *p* = 0.048; filled diamonds), but not ND group (*r* = −0.009, *p* = 0.975; clear squares).

To attempt to further understand the nature of these differences, correlations were performed between activity during end-point and one-step queries in the regions showing a significant difference between groups, and performance on the task (Figure 5). A significant negative correlation was found in the D group between activity and response times to end-point queries (*r* = −0.579, *p* = 0.048). The longer the response time, the less activity was found in the parietal regions showing a difference between groups. As Figure 5 shows, this correlation was only found in the D group, with no such relationship in the ND group (*r* = −0.009, *p* = 0.975).

Given the difference in the parietal cortex response between groups during the test phase, further *post hoc* analyses were conducted in order to test for differences during the learning phase in this parietal region. Two separate masks of the parietal activation showing differences between the D and ND groups in response to one-step and end-point queries were created. In two separate analyses, these were inputted into the learning phase group feat analysis using pre-threshold masking. For both types of queries (one step and end point), the D group do show significant activation in the corresponding parietal region during the learning phase, whereas the ND group do not. The contrast between the two groups (D–ND) does show a significant difference in this region of the parietal cortex during the learning phase (*x* = 48, *y* = −28, *z* = 40, *Z* = 3.5). The same activation peak during the learning phase was seen using the one-step and end-point parietal cortex masks, as these masks almost entirely overlap.

## Discussion

Our results indicate that linear-order reasoning is an effective strategy when learning about, and reasoning with, naturalistic orders in humans. Moreover, hippocampal, parietal, and prefrontal cortical activations during the task provide corroborative evidence for a network of regions associated with reasoning found in previous studies (Christoff et al., 2001; Acuna et al., 2002; Knauff et al., 2002; Goel and Dolan, 2004; Schubotz et al., 2004; Fangmeier et al., 2006; Greene et al., 2006; Wendelken et al., 2008). In accordance with our hypotheses, greater activation was shown by the mildly depressed group compared to the ND group in spatial areas supporting transitive reasoning, namely the parietal cortex, during the spatial-like operations of solving the reasoning queries. This may be a compensatory mechanism in order to reach the same behavioral performance as the ND group [see Thomas and Elliott (2009), Brzezicka (2013)], or evidence for a different reasoning strategy in the depressed group. In *post hoc* analyses, corresponding differences in parietal activation between the two groups were also found for the learning phase.

### Depressed group show relatively greater parietal activation during reasoning

When solving the test queries, the depressed group showed relatively greater activation in the superior parietal lobe and in the region of the supramarginal gyrus and post-central gyrus compared to the ND group who showed relative deactivation during the task (relative to baseline and the depressed group). Activation in the somatosensory cortices (post-central gyrus) is assumed to reflect movement or non-task-related sensory feedback from pressing the response button, in line with suggestions by Acuna et al. (2002).

The greater activation in the parietal cortex during the test phase in the depressed group may be more task-related. The parietal lobe has been shown to be involved during mental operations that require spatial manipulation of internal representations, such as transitive inference (Goel and Dolan, 2001; Acuna et al., 2002; Knauff et al., 2002; Monti et al., 2007). Recently, Waechter et al. (2012) showed that patients with focal lesions in the parietal cortex were significantly impaired on transitive reasoning tasks, as compared to normal controls. It appears that the depressed group required more activation than the ND group at test to make the spatial aspects of the task sufficiently salient to arrive at the same behavioral outcome. It should be noted, however, that by using the contrast with fixation to examine the between group differences, this interpretation is not the only one possible. Greater parietal lobe activation between depressed and ND in the particular contrast D(test-fixation) − ND(test-fixation) could either reflect greater parietal lobe activation during the task (as stated), but alternatively could reflect no change in task activation but a greater deactivation during fixation in the depressed relative to the ND. Future research should further examine these possibilities.

The longer the time the depressed group took to respond to the test queries, the less activation was found in the parietal cortex. In other words, the quicker the depressed individuals responded, the more effort was indicated by brain activation. Given that this correlation is based only on correct responses, it appears that depressed participants needed to spend more effort to achieve quicker, correct responses, a correlation not found in the ND participants. These results are in accordance with earlier behavioral findings of Sedek and Von Hecker (2004). These authors suggested that depressed individuals are not as successful or efficient in constructing a linear order during the learning stage, and so engage in a different, compensatory style of reasoning when prompted by a test query. By compensation we mean that the same region in the brain may have to work harder in the depressed group than in the ND control group, in order to achieve the same performance level. This may be expected if depression is associated with more difficulties in the early deployment of suitable strategies of task execution and information integration (Hertel and Rude, 1991; Sedek and Von Hecker, 2004).

Our argument follows the general logic that processing disadvantages can be indicated by the observation that in order to achieve the same level of performance in a cognitive task, the disadvantaged group (in our case, depressed individuals) has to exert relatively more mental effort than the non-disadvantaged group (ND individuals). As such, this reasoning has previously been applied to other domains within the literature on behavioral correlates of cortical activation. For example, Fangmeier et al. (2006) (Ruff et al., 2003) suggested that for individuals with high spatial ability, the reasoning problems may have required less demand for visuospatial processing such that less activity in the parietal cortex was required to solve the problems, as compared to individuals with low spatial ability. In our case, the relative deactivation shown in the ND group in this study may take this argument one step further. A number of explanations for decreases in the BOLD signal have been put forward, including suppression of task irrelevant activity or reallocation of resources [e.g., McKiernan et al. (2003), Tomasi et al. (2006)], the default mode network (Raichle et al., 2001; Singh and Fawcett, 2008), greater activity in the baseline task than the task of interest (Gusnard et al., 2001; Stark and Squire, 2001), or optimizing activity to focus task performance (Astur and Constable, 2004; Rekkas et al., 2005). It is possible that the deactivation seen in the ND group could be explained as optimization of the activity in the parietal cortex, along the lines of that suggested for hippocampal deactivation during a similar relational task (Astur and Constable, 2004), in which it was suggested that inhibition was used to dampen irrelevant relations while the representation of important relations remained. This would be in line with the behavioral data, which suggests that retrieval of the correct response is made easier through the use of an organized mental array [see also Leth-Steensen and Marley (2000), Sedek and Von Hecker (2004)]. It is possible that the ND group, after successful construction of a mental array, tend to inhibit any additional (i.e., unnecessary) spatial processing that could interfere with retrieval from the already existing representation.

The fact that in the *post hoc* analyses, the depressed, unlike the ND, group displayed significant activation levels in the target parietal region during the learning phase may be due to the characteristics of the assumed process of mental model construction. As argued earlier (Sedek and Von Hecker, 2004), depressed individuals may find such construction more difficult to do than ND individuals. If it is further assumed that construction takes place in the learning phase, and that spatial functions are involved in this type of construction (Leth-Steensen and Marley, 2000), the more intense recruitment of parietal regions in the depressed group during learning appears plausible. It is further plausible to speculate that depressed individuals, more so than ND participants for whom construction would be easier (and already accomplished at the time of testing), would again recruit parietal regions more, even at test, in their attempts to arrive at clear mental models of the rankings^2^.

While differential brain activity was found between groups during the test phase and the learning phase, the behavioral results, and the debriefing following scanning, did not show significant differences in performance between the depressed and the ND group, in contrast to earlier findings (Sedek and Von Hecker, 2004). The difference in the results between this study and these earlier findings could be due to differences in the paradigm arising from changes needed to prepare the task for fMRI; for example, participants were given extensive practice up to a criterion before being admitted to the task, unlike in Sedek and Von Hecker (2004), so the lack of performance differences may be due to a ceiling effect. Also, the timing in the fMRI task provided participants with a fixed study time of 10 s when learning the relations as opposed to response-driven timing, thereby providing more structure to the task, and possibly helping to focus attention. Indeed, Hertel and Rude (1991) showed that depressed participants exhibited performance deficits only in task conditions where their attention remained unfocused during task execution, but had normal performance when their attention was focused by task constraints.

This discrepancy between the group differences showing the neuroimaging results but not the behavioral data is not unprecedented. There is evidence to suggest that there are cognitive impairments in depression that are only demonstrable using neuroimaging techniques. Several studies have shown comparable performance on working memory and Stroop interference tasks in depressed and control participants, but in association with increased activation of the PFC in the depressed group (Wagner et al., 2006; Matsuo et al., 2007; Walter et al., 2007). Explanations for this differential brain activation include compensatory recruitment of PFC resources to complete the task successfully (Walter et al., 2007) and cortical inefficiency due to hyperactivity of key brain regions (Wagner et al., 2006). Smith et al. (2014) induced effect in a within-participant design by having participants view positive, negative, and neutral picture stimuli. They found that emotion did not impair logical reasoning, but that the neural systems underlying such reasoning differed in activation from those in the neutral condition. This dovetails with our finding that equivalent levels of reasoning between depressed and ND participants were associated with different activation levels in brain areas known as underlying performance in the particular task.

### Greater prefrontal activation during inference

Several studies now suggest that the rostral PFC is important for integration of relations into an internal representation (Christoff et al., 2001; Kroger et al., 2002; Fangmeier et al., 2006; Van Opstal et al., 2008; Wendelken et al., 2008). The results from the ND group in this study clarify this further by suggesting that rostral medial PFC (BA 8 and 10) activity is required when making novel inferences by manipulating information within an integrated mental model compared to recalling the answer to queries on previously presented relations. While some studies have found lateral RPFC activity to be associated with relational integration (Christoff et al., 2001; Wendelken et al., 2008), others have found medial RPFC activation, including the present one (Fangmeier et al., 2006; Van Opstal et al., 2008). In a review of models into the functions of the anterior PFC (BA 10), Ramnani and Owen (2004) suggest that the role of this region overall is “in integrating outcomes of two or more separate cognitive operations in the pursuit of a higher behavioral goal” (p. 1). The exact location of the activation found could be a function of the particular task employed, the specific cognitive processes required, sample recruited, stimuli used, and so on.

### Limitations and Conclusion

These results should be considered in light of the limitations of the study. The study was designed to compare directly activation between test queries or the learning phase, as well as between groups, as such a fixation baseline was deemed adequate. More specific findings relating to the learning phase, in particular, may have been possible with a baseline that provided greater control over the non-reasoning task processes, such as reading or making a response. Also we were unable to differentiate between activation associated with maintaining the structure of the array (ABCD) when presented in correct order type (i), as compared to the shuffled order type (ii) which should pose greater integration demands. These cognitive demands appeared not to require differential brain activity within this design. However, this investigation may have been improved if the design had allowed a greater number of examples of each type.

In conclusion, we have shown that reasoning with naturalistic linear orders in humans is subserved by a similar network of brain regions, including hippocampus, parietal, and prefrontal cortices, as compared to reasoning with purely abstract information found in previous studies. As predicted, sub-clinically depressed participants demonstrated higher activation of parietal areas during a test, and the learning, of presented and inferred relations, possibly reflecting a different strategy of task execution.

## Conflict of Interest Statement

The authors declare that the research was conducted in the absence of any commercial or financial relationships that could be construed as a potential conflict of interest.

## Supplementary Material

The Supplementary Material for this article can be found online at http://www.frontiersin.org/Journal/10.3389/fnhum.2014.01061/abstract

## Footnotes

^1^We thank the reviewer for pointing out that the inferred vs. presented contrast can be difficult to interpret since the recall can interfere with the model creation and the inference process. Maybe the participants hold the premises in mind and rehearse them more or less extensively, which may interfere with later stages of model creation.

^2^We thank the reviewer who drew our attention to the possibility that part of the reason why such parietal recruitment may be particularly required in depressed individuals may be the fact that the premises for model construction, i.e., the one-step pairs, are still rehearsed at test in the depressed, which would potentially entail ongoing constructive effort during test, and as such would interfere with their quick and efficient use of the mental model as a retrieval device. We agree. This possibility is in line with earlier research showing that in non-depressed individuals, premises of transitive mental models tend to be forgotten after successful construction (Mayberry et al., 1986), and that sad and depressed individuals tend to process detail information meticulously, i.e., preserve behavioral information more than individuals in neutral mood, when inferences from that information can be drawn (Gannon et al., 1994; Yost and Weary, 1996).
