# Supplementary material for: Reasoning with Linear Orders: Differential Parietal Cortex Activation in Sub-Clinical Depression. An fMRI Investigation in Sub-Clinical Depression and Controls
Source: Front Hum Neurosci. 2015 Jan 19;8:1061. doi: 10.3389/fnhum.2014.01061 (PMC4298224; doi:10.3389/fnhum.2014.01061)
Supplement: Supplementary file 1 [file Table_1.PDF]

APPENDIX

Table S1: Activation loci associated with the learning phase

Region	Hemisphere	z value	co-ordinates		
			x	y	z
Frontal pole	L	5.8	-50	46	-12
Frontal pole (BA 10)	R	5.7	40	42	20
Frontal pole (BA11)	L	5.02	-18	62	-16
Middle frontal gyrus (BA46)	L	6.22	-50	26	30
Superior frontal gyrus (BA6)	L	7.39	-2	4	56
Superior frontal gyrus	L	6.36	-30	0	68
Middle/superior frontal gyrus	R	6.2	30	8	60
Insular cortex (BA13)/frontal orbital cortex	L	6.56	-36	18	-6
Insular cortex/frontal orbital cortex	R	7.12	34	20	-6
Inferior frontal gyrus, pars triangularis	L	5.03	-36	24	20
Temporal pole		5.11	-52	14	-10
Precentral gyrus	L	6.75	-38	-4	40
Precentral gyrus (BA6)	L	5	-62	6	12
Pallidum / amygdala	L	6.67	-20	-14	-12
Pallidum	R	5.42	14	-2	-4
Hippocampus	L	7.02	-26	-32	-10
Hippocampus	R	6.88	24	-32	-10
Supramarginal gyrus (parietal lobe) BA40	L	6.25	-44	-48	36
Supramarginal gyrus (parietal lobe) BA40	R	6.64	46	-42	46
Lateral occipital cortex/parietal lobe (BA7)	L	6.91	-28	-68	50
Lateral occipital cortex/parietal lobe	R	6.41	32	-66	52
Lateral occipital cortex/parietal lobe	L	5.96	-28	-72	38
Lateral occipital cortex/parietal lobe	R	6.96	30	-66	30
Precuneus		6.27	0	-76	54
Lingual gyrus	R	7.35	12	-90	-10
Lingual gyrus	L	7.2	-14	-88	-16
Cerebellum	L	7.71	-14	-84	-28
Cerebellum	R	7.4	24	-82	-30
