# Supplementary material for: Reasoning with Linear Orders: Differential Parietal Cortex Activation in Sub-Clinical Depression. An fMRI Investigation in Sub-Clinical Depression and Controls
Source: Front Hum Neurosci. 2015 Jan 19;8:1061. doi: 10.3389/fnhum.2014.01061 (PMC4298224; doi:10.3389/fnhum.2014.01061)
Supplement: Supplementary file 2 [file Table_2.PDF]

Table S2: Activation loci associated with the test phase

Region	Hemisphere	z value	co-ordinates		
			x	y	z
**End-point queries - fixation (e.g. A > D)**					
Frontal orbital cortex / inferior frontal cortex	L	7.17	-36	14	10
Frontal orbital cortex / inferior frontal cortex	L	7.13	-34	24	-14
Frontal orbital cortex / inferior frontal cortex	R	6.90	28	26	-14
Inferior frontal gyrus / precentral gyrus	R	5.86	44	4	20
Supplementary motor area	L	7.11	-8	6	50
Superior frontal gryus (BA6)	R	5.70	28	4	58
Middle frontal gyrus (BA9)		5.73	46	28	26
Hippocampus	R	6.63	24	-30	-8
Hippocampus	L	6.28	-20	-32	-14
Thalamus	L	7.56	-14	-16	4
Supramarginal gyrus / inferior parietal lobule (BA40)	R	7.25	42	-46	44
Superior parietal lobule	R	6.47	36	-52	42
Precuneus (BA7)	R	6.57	8	-74	42
Occipital pole / Lingual gyrus		7.29	12	-90	-10
Occipital cortex	L	7.32	-14	-90	-16
Cerebellum	R	7.29	20	-48	-34
Cerebellum	L	7.26	-14	-84	-28
**Two-step queries - fixation (e.g. A > C)**					
Frontal pole (BA9)	R	5.77	44	40	28
Frontal orbital cortex (BA47) / Insula	L	7.00	-34	18	-6
Frontal orbital cortex / Insula	R	7.04	26	24	-8
Supplementary motor area	L	7.07	-6	6	56
Precentral gyrus	L	7.01	-36	-10	56
Thalamus	R	6.64	12	-8	2
Thalamus	L	7.08	-10	-14	4
Parahippocampal gyrus (BA35)	L	7.04	-22	-30	-16
Hippocampus	L	6.39	-24	-32	-6
Hippocampus	R	6.53	24	-30	-6
Supramarginal gyrus (BA40)	L	7.02	-44	-40	38
Supramarginal gyrus (BA40)	R	6.65	40	-46	40
Precuneus	R	6.21	6	-74	42
Lateral occipital cortex	R	6.18	30	-66	32
Lateral occipital cortex / precuneus	L	6.96	-28	-66	46
Occipital fusiform gyrus	L	7.29	-14	-90	-16

Cerebellum	L	7.37	-14	-86	-28
Cerebellum	R	7.10	12	-86	-10
**One-step queries - fixation (e.g. A > B)**					
Frontal orbital cortex / Insula	R	7.22	26	22	-6
Frontal orbital cortex / Insula	L	6.63	-32	22	-8
Frontal pole (BA10)	L	5.37	-32	48	20
Supplementary motor area	R	7.34	-8	6	50
Precentral gyrus	R	7.34	-36	-4	42
Medial frontal gryrus (BA6)		7.09	0	16	44
Middle frontal gyrus	R	5.39	36	4	38
Post central gryus (BA5)	L	5.15	-40	-40	66
Thalamus	L	7.76	-12	-14	2
Thalamus	R	6.69	12	-8	0
Hippocampus / Parahippocampal gyrus (BA27)	L	7.14	-20	-32	-6
Hippocampus	R	6.69	22	-30	-8
Heschl's gyrus / central operculum cortex	L	5.78	-48	-22	12
Occipital lobe / Lingual gyrus	R	7.51	12	-86	-10
Occipital lobe / Lingual gyrus	L	6.98	8	-76	2
Cerebellum	L	7.24	-14	-86	-28
